# Increased Uptake of Chelated Copper Ions by *Lolium perenne* Attributed to Amplified Membrane and Endodermal Damage

**DOI:** 10.3390/ijms161025264

**Published:** 2015-10-23

**Authors:** Anthea Johnson, Naresh Singhal

**Affiliations:** Department of Civil and Environmental Engineering, University of Auckland, Auckland 1142, New Zealand; E-Mail: a.johnson@auckland.ac.nz

**Keywords:** phytoextraction, amendment, model, chelator, mechanism, plant, membrane, permeability

## Abstract

The contributions of mechanisms by which chelators influence metal translocation to plant shoot tissues are analyzed using a combination of numerical modelling and physical experiments. The model distinguishes between apoplastic and symplastic pathways of water and solute movement. It also includes the barrier effects of the endodermis and plasma membrane. Simulations are used to assess transport pathways for free and chelated metals, identifying mechanisms involved in chelate-enhanced phytoextraction. Hypothesized transport mechanisms and parameters specific to amendment treatments are estimated, with simulated results compared to experimental data. Parameter values for each amendment treatment are estimated based on literature and experimental values, and used for model calibration and simulation of amendment influences on solute transport pathways and mechanisms. Modeling indicates that chelation alters the pathways for Cu transport. For free ions, Cu transport to leaf tissue can be described using purely apoplastic or transcellular pathways. For strong chelators (ethylenediaminetetraacetic acid (EDTA) and diethylenetriaminepentaacetic acid (DTPA)), transport by the purely apoplastic pathway is insufficient to represent measured Cu transport to leaf tissue. Consistent with experimental observations, increased membrane permeability is required for simulating translocation in EDTA and DTPA treatments. Increasing the membrane permeability is key to enhancing phytoextraction efficiency.

## 1. Introduction

Metal-chelating amendments have previously been investigated for their ability to increase metal translocation to aerial plant tissues, in order to increase the rate of phytoextraction from contaminated soil [1,2,3,4]. They have previously been shown to alter Cu distribution at a range of scales within plants [5,6]. These include alterations in distribution between mobile and sorbed phases, with chelated Cu ions found to have greater mobility than free ions. Chelates have also been found to alter metal distribution within root tissue, increasing Cu levels within the central stele compared to outer cortical tissue [7,8]. Chelated metals are transported more readily to shoot tissue than free metal ions [9,10,11], with higher root-to-shoot translocation coefficients that are not proportional to transpiration rates. In addition to influencing metal transport, chelating agents have been found to alter plant physiology, increasing membrane permeability [12,13] while reducing Cu-induced peroxidative damage [7]. Multiple pathways exist for root-to-shoot metal transport [14]: the extracellular apoplastic pathway; the intracellular symplastic pathway; and the transcellular pathway that involves multiple membrane transport steps. Different transport pathways are postulated for free and chelated metal ions. Transport of free metal ions is hypothesized to occur via the apoplastic pathway across the root cortex, with apoplastic bypass flow through the endodermis facilitated by endodermal damage [15]. Chelation of metal ions is hypothesized to facilitate symplastic transport across the cortex by increasing membrane permeability [9] and reducing apoplastic bypass flow through the endodermis. A number of mechanisms are implicated, though the relative contributions of these to the overall rate of metal translocation are unclear. A quantitative assessment of these mechanisms would allow identification of the limitations to metal translocation in plants, potentially enabling these to be addressed.

Modelling has been used to identify and test the limiting mechanisms and processes controlling metal uptake in plants [16]. These may include the influence of transpiration rate [17], the sorption of solutes to soil or plant tissues [18], the effects of amendment and contaminant-induced damage to cell membranes and other tissues [19], and the effect of tissue permeability to water and solutes [20]. Models have previously been developed to describe the flow of water and movement of organic and inorganic substances in plant tissues [21,22] and the soil of the root zone (the rhizosphere) [23,24,25]. Existing models cover a range of scales, from the field scale [26] to the subcellular level [27]. Few models address the influence of chelators on metal transport within plant tissues, and the root is often regarded as a passive solute sink [23,28,29,30].

Here we aim to identify mechanisms for chelate-enhanced phytoextraction through quantitative assessment of the processes influenced by chemical amendments. These include transpiration rate, apoplastic sorption, endodermal development, membrane permeability and diffusion (both apoplastic and symplastic). For this purpose, we developed a plant-scale model of the processes controlling water and metal movement within plant tissues. Following identification of key parameters using sensitivity analysis, we performed simulations against experimental results to identify the mechanisms influenced by the addition of chelating amendments.

## 2. Results and Discussion

### 2.1. Identification of Critical Parameters Using Sensitivity Analysis

Sensitivity analysis using Latin Hypercube Sampling (Table A1) indicated that the most influential model parameters are the diffusion coefficients in the apoplast (*D*_a_) and symplast (*D*_s_), the apoplastic sorption coefficient (*K*_L_), endodermal integrity (*EDF*) and membrane permeability (*P*_m_). Of the seven global parameters tested, those found to significantly (*p* < 0.05) influence Cu concentrations within the plant are *EDF*, *P*_m_, *K*_L_ and *D*_a_. Solute dispersivity (α) and flow velocity (*v*_factor_) do not significantly alter Cu levels or distribution in the plant. The influence of both *D*_s_ and *EDF* is relatively small, although *EDF* significantly influences root cortical concentrations. The influence of both *D*_a_ and *K*_L_ on root-to-shoot translocation is significant, while that of *P*_m_ is highly significant (*p* < 0.001).

Relatively high or low values for each of these parameters are likely to support either apoplastic or symplastic transport. A high ratio of apoplastic to symplastic diffusivity is likely to favor transport via the apoplastic route, due to lower diffusive resistance, while the converse will be true if the ratio is reversed. Higher apoplastic sorption is expected increase the fraction of Cu retained in the apoplast. Endodermal damage will increase the apoplastic fraction of both water and solute transport, while increased membrane permeability will encourage solute entry to the symplastic pathway.

### 2.2. Simulations

**Figure 1:**
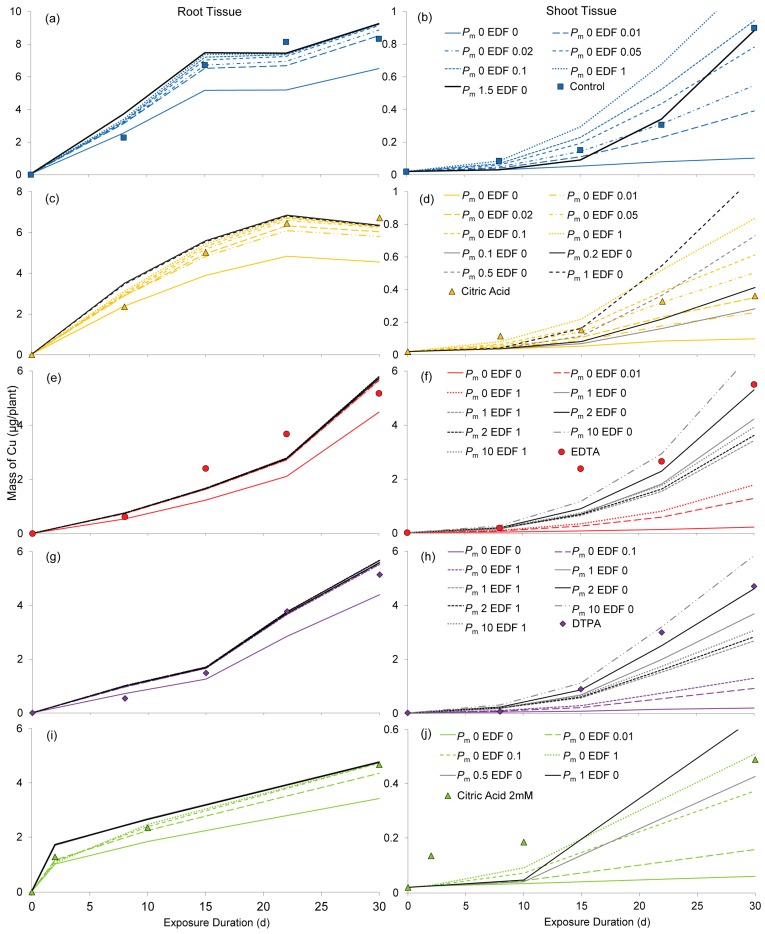
Simulations of total Cu mass (µg/plant) in plant tissues calibrated against experimental results for amendment treatments: (**a**) control roots; (**b**) control shoots; (**c**) 0.157 mM citric acid roots; (**d**) 0.157 mM citric acid shoots; (**e**) EDTA roots; (**f**) EDTA shoots; (**g**) DTPA roots; (**h**) DTPA shoots; (**i**) 2 mM citric acid roots; (**j**) 2 mM citric acid shoots Lines show simulations with varying membrane permeability (*P*_m_) and endodermal damage fraction (*EDF*) for simulated amendment treatments. Points without lines show experimental data presented previously [7,45].

Model parameters are calibrated using experimental data for the amendment treatments and presented in Figure 1. Simulations for the control (+Cu) treatment (Figure 1a,b) show that translocation to shoots can occur entirely apoplastically (*P*_m_ = 0) if the endodermal barrier is damaged. Endodermal damage was estimated at 5% (range 2%–10%). If no endodermal damage is assumed, membrane permeability of 1.5 m/day is required to allow sufficient solute transport via the symplastic pathway to support the observed translocation rate; however this results in somewhat delayed uptake. Root Cu accumulation was calibrated using an apoplastic sorption coefficient of 0.0015, which is nearly two-thirds less than the estimated value, and roughly half of that derived previously [31].

Transport of Cu with 0.157 mM citric acid can also be explained by a purely apoplastic pathway (Figure 1c,d). The sorption coefficient calibrated with the data for the citric acid treatment had a value of 0.00085, which is slightly over half of the value derived from experimental data. The proportion of endodermal damage is likely to be around 5%, (range 2%–10%), equivalent to that seen for simulations of the control (+Cu) treatments. If the endodermis is intact (*EDF* = 0), membrane permeabilization can also support observed translocation, with *P*_m_ < 0.5 m/day. However, increasing the symplastic transport component causes a delay in Cu translocation to shoot tissue.

Purely apoplastic treatment is insufficient to describe Cu translocation in EDTA treatments (Figure 1e,f), even when the endodermal barrier is removed entirely (*EDF* = 1). Membrane permeability must be greater than 2 m/day, which is approximately four times the average of reported values for organic solutes [32,33,34,35], and orders of magnitude greater than those for inorganic solutes [21,32,36,37,38,39,40,41,42,43,44]. Endodermal damage appears to lower Cu accumulation in shoots, requiring higher permeability values to simulate experimental data. The calibrated sorption coefficient is 25% higher than that predicted from earlier experiments (using dead tissue), which may indicate that some Cu is absorbed by the symplast.

Results for DTPA (Figure 1g,h) are remarkably similar to those for EDTA, with a symplastic transport component required to fit experimental data for shoot tissue. Membrane permeability must be greater than 2 m/day, and higher still if the endodermis is damaged. The sorption coefficient (*K*_L_ = 0.0002) estimated by calibration with experimental data is a third higher than that obtained from sorption experiments.

When the endodermis is completely damaged or undeveloped (*EDF* = 1), transport by a purely apoplastic pathway (*P*_m_ = 0) can account for observed Cu accumulation in shoot tissue of plants in the citric acid 2 mM treatment (Figure 1i,j). Alternatively, if no endodermal damage is assumed, an increase in membrane permeability (*P*_m_ between 0.5 and 1 m/day) will raise shoot Cu levels to approximate those observed in the experiments. This however induces a further lag in translocation, which adds to that already observable in simulations of the apoplastic pathway (*P*_m_ = 0). The calibrated sorption coefficient is 0.0009, a value 55% greater than predicted.

Combinations of parameter values that were found to best fit the experimental data are presented in Table 1. For the control and citric acid treatments, both apoplastic and symplastic pathways can describe the observed results, whereas symplastic transport is required to describe uptake in the treatments with strong chelating agents (EDTA and DTPA). Speciation data (presented in Table A2) indicates both free and complexed Cu species exist in control and 0.157 mM citric acid treatments, thus the implication of a combination of pathways is not unexpected.

**Table 1:** Values of sensitive parameters for apoplastic diffusion (*D*_a_), sorption (*K*_L_), endodermal damage (*EDF*) and membrane permeability (*P*_m_) calibrated with experimental data. For the uptake of Cu in Control and Citric acid treatments, both apoplastic and symplastic pathways are possible, while EDTA and DTPA treatments require symplastic transport.

Treatment	Pathway	Fitted Parameters			
		*D*_a_ (m^2^/day)	*K*_L_ (m^3^/g)	*EDF*	*P*_m_ (m/day)
Control	Apoplast	3.1 × 10^−5^	0.0015	0.05	0
	Symplast			0	1.5
Citric acid (0.157 mM)	Apoplast	1.7 × 10^−5^	0.00085	0.05	0
	Symplast			0	0.4 (0.2–0.5)
Citric acid (2 mM)	Apoplast	1.4 × 10^−5^	0.0009	1	0
	Symplast			0	0.6 (0.5–1)
EDTA	Symplast	1.2 × 10^−5^	0.0002	0	2
DTPA	Symplast	0.9 × 10^−5^	0.0002	0	2

### 2.3. Evaluation of Simulated Transport Mechanisms

#### 2.3.1. Sorption

As can be seen in Figure 1, alterations to sorption in the cortical apoplast greatly influence shoot Cu accumulation. Decreased apoplastic sorption allows higher solute mobility, with higher translocation rates observed. Calibrated sorption coefficients are 63% and 47% lower than predicted for control and citric acid simulations respectively, but 25% and 33% higher respectively than predicted for EDTA and DTPA. The calibrated value for the high level (2 mM) citric acid treatment is 55% higher than predicted. These results may indicate that some solute retention in the symplast occurs for chelate treatments, perhaps due to membrane permeabilization, or increased metal sequestration in the vacuole. Sorption coefficients were estimated from experiments with dead tissue, where membrane partitioning processes would be neglected. Lower sorption coefficients for the control and citric acid treatments may be due to endogenous production of chelating compounds, which could be expected to reduce Cu sorption. These would also have been unaccounted for in sorption experiments with dead tissue.

#### 2.3.2. Endodermal Damage

**Figure 2:**
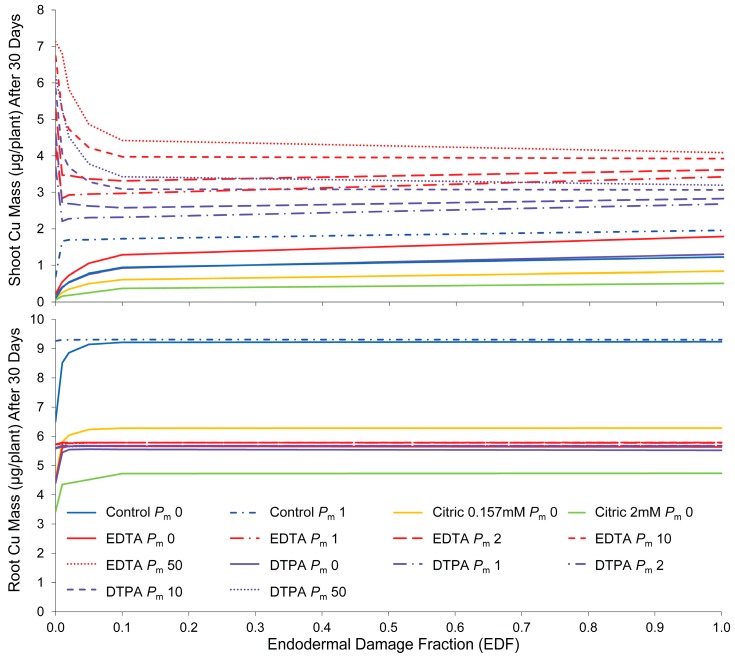
Influence of endodermal damage on simulated Cu accumulation by root and shoot tissue in control and chelator treatments, with *P*_m_ indicating varying levels of membrane permeability, from impermeable (*P*_m_ 0) to highly permeable (*P*_m_ 50).

As shown in Figure 2, when solute transport occurs by the purely apoplastic pathway (*P*_m_ *=* 0), accumulation of Cu in root tissue is sensitive to small changes in endodermal integrity in the range below 10%. Above this range, alterations in *EDF* have little influence on root Cu levels. This range drops to less than 1% in cases where symplastic transport is included by increasing membrane permeability.

Endodermal damage strongly influences Cu accumulation in shoot tissue. When membrane permeability is low, endodermal damage increases shoot Cu accumulation due to higher apoplastic bypass flow. This shows that the endodermis can control apoplastic solute translocation to leaf tissue. However, the apoplastic pathway provides higher resistance to solute movement, and solute transport by this route alone cannot fully account for high Cu accumulation recorded in the experiments with chelator treatments. Where membrane permeability is incorporated to allow transport by the symplastic pathway, increased *EDF* actually decreases shoot Cu accumulation, by reducing symplastic flow and therefore the movement of ions into the xylem. Membrane damage (increased permeability) has a much greater influence on shoot concentrations if endodermal damage is low.

#### 2.3.3. Membrane Permeability

Membrane permeability determines the distribution between apoplastic and symplastic transport across the root cortex. Apoplastic transport is sufficient to describe translocation in control and 0.157 mM citric acid treatments, provided the endodermal barrier is damaged or otherwise undeveloped. However, for treatments with strong chelating agents (EDTA and DTPA), less than half of the translocation to shoots can be explained by a purely apoplastic pathway, even in the complete absence of the endodermal barrier. The other component is likely due to symplastic transport, requiring movement across the plasma membrane. Estimated membrane permeabilities are conspicuously different from those obtained by calibration of the models with experimental data. Although there is considerable variation between permeabilities obtained by the different estimation methods (Table A3), control (+Cu) and citric acid treatments were both expected to have relatively high membrane permeabilities compared to those for treatments with chelating agents. Calibration with experimental data found the opposite trend, indicating that the chelating agents have a permeabilizing influence. The effect of increasing membrane permeability becomes asymptotic at high levels, showing the limitation of the symplastic pathway.

As the hydrophilic chelates are unlikely to diffuse directly through the lipid bilayer, it is assumed that they have a permeabilizing effect on the membrane via an unknown mechanism. Lipophilic diffusion and lipid peroxidation are unlikely to account for these effects, as shown previously [7]. Potential mechanisms for increased membrane permeabilization include alterations to membrane fluidity, repair and destabilization.

**Figure 3:**
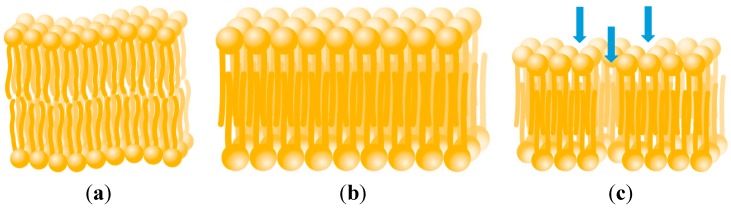
Bilayer structure of phospholipid membrane (**a**); Exposure to Cu^2+^ reduces membrane fluidity resulting in densely-packed lipid regions [46] (**b**); and can also cause the formation of pores [55], indicated by blue arrows (**c**).

The presence of Cu^2+^ ions may increase the permeability of membranes above the level normally expected for healthy cells. Exposure to metal ions can lower the membrane fluidity, resulting in more densely packed lipid regions [46,47] (Figure 3). Reduced membrane fluidity decreases the permeability of charged or polar substances by lowering solute diffusion through the lipid bilayer and transient aqueous cavities [48]. However, increased membrane rigidity has been reported to correlate with cell leakiness, a measure of toxicity [49]. This has been found to be partially ameliorated by the exudation or exogenous addition of organic acids (including citric acid) and other ligands such as EDTA [50,51,52]. Increased membrane lipid rigidity can increase the permeability of non-electrolytes through the lipid regions [53,54], although reductions have also been observed (possibly due to bilayer asymmetry) [48]. Exposure to Cu^2+^, in addition to reducing membrane fluidity [46], can also result in the formation of pores in the membrane (Figure 3), increasing non-specific permeability [55]. This may result in higher permeabilities than typically reported for healthy membranes. EDTA has previously been observed to influence membrane integrity [56]. It has been found to increase membrane permeability when present in conjunction with boric acid [57], but not citric acid [58]. This is thought to be due to synergistic effects of EDTA and boric acid resulting in increased membrane fluidity [58].

**Figure 4:**
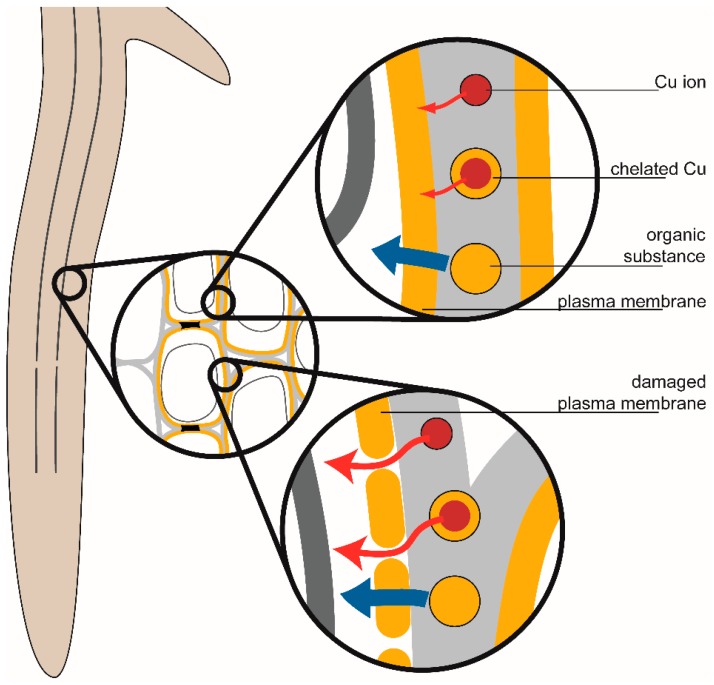
Influence of membrane damage on permeability to ions and organic molecules. Lipophilic partitioning of organic substances occurs through lipid regions, while charged species diffuse slowly through transient aqueous cavities and, in damaged membranes, pores formed due to Cu^2+^ exposure.

Chelating agents may indirectly have a permeabilizing influence on cell membranes by preventing repair of the plasma membrane [9]. Divalent cations such as Ca^2+^ and Zn^2+^ act to close pores formed by cytotoxic agents [59,60,61]. However, the chelation of these ions by citric acid, EDTA, and DTPA may prevent their involvement in this process. Increased membrane permeability to organic compounds and metal ions are due to membrane damage are illustrated in Figure 4.

Strong chelating agents such as EDTA can destabilize membranes by chelating divalent metals from lipopolysaccharides and membrane-associated proteins [62,63,64,65] resulting in a loss of material from the membrane, and increased permeability [66,67]. The permeability of membranes exposed to 0.18 mM EDTA (a similar concentration to that used in this research) was found to be 27-fold higher than control treatments, while 1.2 mM citrate had no permeabilizing effect [68]. Vassil *et al.* [9] noted the higher damaging effect of free EDTA compared to EDTA chelates, with the finding that the addition of equimolar Pb^2+^ resulted in lower foliar necrosis than that induced by EDTA alone. They speculated that toxicity was due to binding of membrane-stabilizing divalent cations by uncoordinated EDTA [9]. This contrasts with the findings presented previously [7], where chelating agents appeared to be less damaging alone than when applied with equimolar Cu. The related chelating agent ethylenediamine-*N*,*N*′-disuccinic acid (EDDS) has also been found to significantly increase both membrane permeability and cytoplasmic Cu levels, resulting in higher Cu accumulation in shoot tissue [12]. Organic acids including citric acid have previously been found to increase plasma membrane permeability to a greater extent than inorganic acids [69]. This indicates that factors other than solution acidity are likely to be responsible for membrane permeabilization.

#### 2.3.4. Diffusion

Simulating increased diffusion in the cortical apoplast was observed to increase Cu accumulation in both root and shoot tissue. This was due to increased transport rates, rather than tissue capacity for Cu. In roots, increased diffusion decreases the time until equilibration (concentration plateau). In shoots, higher diffusion decreases the time required for Cu to start accumulating in tissues.

### 2.4. Evaluation of Transport Pathways

For each hypothesized transport pathway, the existence and contribution of supporting mechanisms were assessed based on their support from the literature, experiments, and modelling. In some cases, conflicting results were obtained from the literature and experiments regarding the existence of potential mechanisms and their effect on Cu translocation. The model was used to evaluate the ability of the apoplastic and symplastic pathways to account for the transport of Cu and Cu-chelates, respectively, across the endodermis, which is assumed to represent the main barrier to solute movement in the plant.

#### 2.4.1. Apoplastic Transport of Cu^2+^

The model simulations indicate that purely apoplastic transport can describe Cu translocation in the control treatment. Apoplastic transport is simulated using a combination of high sorption, endodermal damage and apoplastic diffusion, and low membrane permeability. Endodermal damage is the most critical mechanism controlling transport of free Cu ions via the apoplastic pathway. This may be caused by levels of Cu exposure that result in cell death. Severe membrane damage that triggers plasmolysis will cause affected cells to lose their cytoplasmic (symplastic) component and become part of the apoplastic pathway. As this occurs, plasmodesma connections are sealed off [70], maintaining the integrity of the membrane-bound symplastic continuum. If Cu-induced peroxidative damage is severe, plasmolyzed endodermal cells may provide sites for ready ingress of solutes to the stele. Deposition of additional suberin barrier material would be necessary to repair this damage, preventing apoplastic bypass flow. This is supported by energy-dispersive X-ray spectroscopy results where increases in endodermal silicon (linked to suberin deposition) were observed over time [7]. The model results lend further support to the hypothesized apoplastic bypass of Cu through the endodermis.

Alternatively, by assuming a membrane permeability of 1.5 m/day, the transport of free Cu can be modeled with the inclusion of a symplastic transport component. This permeability value is higher than the 0.4 m/day derived for the citric acid treatment, and more comparable to the 2 m/day derived for the EDTA and DTPA treatments (Table 1). In previous experiments [7], Cu increased membrane permeability to nuclear stains, though the effect was noticeably lower than when chelating agents were also present. On these grounds, symplastic transport dependent on a high membrane permeability value for Cu may not be a reasonable explanation for observed translocation in the control treatment.

#### 2.4.2. Symplastic Transport of Cu Chelates

Transport by the symplastic pathway is facilitated by high membrane permeability combined with low sorption, endodermal damage, and apoplastic diffusion. Although there are conflicting findings for the effects of amendments on Cu transport mechanisms and pathways, on balance there is support for chelate-enhancement of transport via the symplastic pathway. The main effect of chelating amendments within plant tissues appears to be increasing Cu-induced permeabilization of the plasma membrane, but the exact mechanism for this requires further investigation. Chelates also have higher symplastic: apoplastic diffusivity ratios than the free ion, which means that the symplastic transport pathway will present a lower resistance route for Cu movement. The effect of decreased apoplastic sorption will increase both apoplastic and symplastic transport.

Other literature supports the symplastic transport hypothesis, although the mechanisms for the effect are not yet well known. Using transmission electron microscopy, Jarvis and Leung [71] found that Pb chelated with EDTA was transported symplastically in root and shoot tissue of *Chamaecytisus proliferus* (L.f.) link ssp. *proliferus* var. *palmensis* (H. Christ) (Tagasaste), while unchelated Pb and Pb complexed by the related chelator H-EDTA appeared to be retained mainly in the apoplast. Fine Pb deposits were observed in mitochondria, chloroplasts and within plasmodesmata [71]. In a recent study with *Zea mays* [12], the chelator EDDS was found to increase cell membrane permeability, with a concomitant increase in shoot concentrations. Although this was attributed to expansion of the apoplastic pathway through injured passage cells [72], rather than to increased solute entry into the symplast, it indicates that strong chelating agents can permeabilize plant cell membranes. This supports the findings of the experiments and model simulations.

#### 2.4.3. Dual Transport Pathways for Partial Cu Chelation

Simulations of the two citric acid treatments (0.157 and 2 mM) can be compared to further explore the apoplastic and symplastic hypotheses. The influence of citric acid on transport pathways can be inferred by comparing the fitted parameters for the two treatments. While sorption and diffusion coefficients are very similar for both citric acid treatments, the fitted values for *P*_m_ and *EDF* indicate that different transport pathways are involved.

The apoplastic transport pathway is sufficient to describe Cu translocation in the 0.157 mM treatment, assuming 5% endodermal damage. However, this fraction (*EDF*) must increase to 100% (Table 1) in order for the purely apoplastic route to account for Cu transport in the 2 mM citric acid treatment, representing the complete absence of apoplastic barriers in the endodermis for this treatment. This extreme scenario is not supported by EDX results [7], which indicated that levels of Si in the endodermis remained high throughout the exposure period. Symplastic transport is therefore required to explain transport for this treatment. Membrane permeabilities of 0.4 and 0.6 m/day were modeled to fit data for the 0.157 and 2 mM treatments respectively. These values are lower than the value of 2 m/day fitted for the EDTA and DTPA treatments in the absence of endodermal damage.

In the 0.157 mM citric acid treatment, only 70% of Cu occurs as the complexed form (Cu-Citrate^−^), with 20% initially present as the free Cu^2+^ ion in solution, while in the 2 mM citric acid treatment, nearly 97% of Cu is present in the chelated form (Table A2). In both citric acid treatments it is likely that the complex will dissociate as the citrate ligand is degraded, both in solution and through metabolic processes within plant tissues. For these treatments, it is theorized that both pathways contribute to Cu transport, with free and chelated Cu ions transported by the apoplastic and symplastic routes respectively. Over time, as a greater fraction of the Cu present occurs in the free ionic form, the relative contribution of the apoplastic pathway will increase. The implication of symplastic transport for chelate species is consistent with the transport pathways suggested for EDTA and DTPA treatments. Ligand degradation is less likely for EDTA and DTPA, which are known to be resistant to degradation.

## 3. Experimental Section

### 3.1. Model Conceptualization

A finite element approach was used to define barriers and pathways for water and solute movement within the plant, differentiating between apoplastic and symplastic transport pathways [73]. The model incorporates a water flow module, which calculates flow velocities in plant regions based on the water potential gradient applied between the leaf and root surface. Darcy’s law is used to model the transpiration of water from roots to shoots via the xylem vessels [74] due to evaporative flux at the leaf surface [75]; the pressure gradient between regions drives the flow [76,77], while hydraulic conductivity regulates the transpiration rate [78].

**Figure 5:**
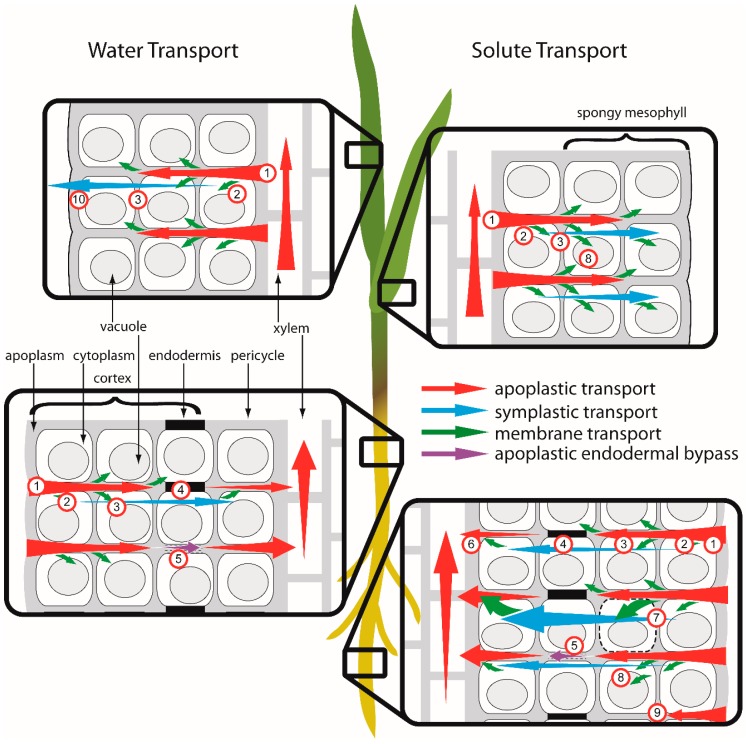
Modeled water and solute transport pathways in root and leaf tissue: (1) apoplastic transport; (2) membrane transport; (3) symplastic transport between cells; (4) symplastic transport across the endodermis; (5) apoplastic endodermal bypass flow; (6) xylem loading; (7) membrane damage; (8) sequestration to vacuole; (9) sorption to apoplastic sites; and (10) evaporation in spongy mesophyll. For clarity, plasmodesmata, which span cell walls and connect the cytoplasm of adjacent cells, are not shown. In root tissue, the endodermis acts as a barrier between cortical and stelar tissues, however, apoplastic bypass flow occurs where the casparian band encircling endodermal cells is damaged or not fully developed.

A coupled solute transport module incorporates advective and dispersive solute movement, membrane transport, vacuolar sequestration and linear sorption. The model simulates a single plant (*Lolium perenne* L.) grown in a solution of uniform and constant concentration, disregarding Cu distribution in soil and the effects of root growth and architecture [79,80,81,82]. The numerical model was constructed using the software FlexPDE^®^ (v6.15, PDE Solutions Inc., Spokane, WA, USA, 2011), which has previously been found to be suitable for modeling solute transport processes in the rhizosphere [83,84,85]. Modeled pathways of water and transport in root and leaf tissue are illustrated in Figure 5.

The model represents a plant symmetrical across the central axis, with the root surface in contact with the bulk solution. Regions modeled include the cortex, endodermis (the inner cell layer of the cortex), stem and leaf. The cortex, endodermis, and leaf contain two homogeneous phases representing the apoplast and symplast separated by the plasma membrane. Zero flux is applied to all boundaries except the outer edge of root and leaf. In the water model, water potential conditions (in the solution and air respectively) are applied to the apoplast at the root edge and the symplast at the leaf edge; a water potential of −100 MPa at 20 °C and 50% relative humidity is assumed in air, and −0.3 MPa in solution at 0.1 m depth [32], approximating conditions used in the experiments [7,86]. In the solute model, a Dirichlet (value) boundary condition is applied at the root edge (apoplast) corresponding to the solute concentration in bulk solution.

### 3.2. Model Simulations

Model simulations tested the possible mechanisms of amendment effects, hypothesized to include reduced Cu sorption to root tissue [7] and alterations to membrane integrity, endodermal development and diffusion. The model is used to simulate results from phytoextraction experiments presented previously [7,45], by calibrating the most sensitive parameters for amendment treatments. The experiments assessed the uptake of Cu (0.157 mM) from hydroponic solution by seedlings of *Lolium perenne* in the presence of chelating amendments including citric acid (0.157 and 2 mM), EDTA (0.157 mM) and DTPA (0.157 mM) [7,45].

Simulations were conducted using the solute model, in conjunction with derived parameter values presented in Table 2. These simulations attempted to simultaneously fit experimental data for Cu accumulation in both root and shoot tissue by adjusting the five most sensitive parameters. Mechanisms controlling uptake and translocation were tested by inspecting the effect of apoplastic sorption (*K*_L_), apoplastic diffusion (*D*_a_), endodermal damage (*EDF*), and membrane permeability (*P*_m_). Due to the relative uncertainty associated with the parameter estimates, an iterative calibration process was applied, progressing from least to most uncertainty (*D*_a_, *D*_s_ < *K*_L_ < *P*_m_, *EDF*). This involved simulating root concentrations by adjusting the sorption coefficient (the most sensitive parameter), followed by increasing membrane permeability and endodermal damage where needed to simulate shoot concentrations. Where *P*_m_ or *EDF* were altered, root concentrations were re-simulated, with adjustments made to *K*_L_ to approximate observed values. Several iterations were used for each amendment.

**Table 2:** Initial estimates of model parameters for calibration with solute model.

Treatment	*D*_s_ (×10^−6^ m^2^/day)	*D*_a_ (×10^−6^ m^2^/day)	*K*_L_ (m^3^/g)	Relative *P*_m_
Control	59	31	0.004	1
Citric acid (0.157 mM)	49	17	0.0016	0.20
EDTA	43	12	0.00016	0.006
DTPA	36	9	0.00015	0.05
Citric acid (2 mM)	47	14	0.00058	–

## 4. Conclusions

Strong chelating agents appear to increase Cu translocation primarily by increasing symplastic transport. The increased translocation observed in amendment treatments can be attributed to a combination of mechanisms. The majority of the effect is due to decreased sorption, with a substantial component of symplastic transport required to fully account for translocation in chelator treatments.

Purely apoplastic transport is able to describe Cu translocation in control and 0.157 mM citric acid treatments, while a symplastic component is required to describe the effects of strong chelating amendments and high concentrations (2 mM) of citric acid. Symplastic transport is increased due to enhanced membrane permeability, although the exact mechanism remains to be elucidated. The ability of chelates to increase membrane permeability is unlikely to be due to lipid peroxidation, nor to increased lipophilicity of the chelate compared to the free metal. Experimental results [7] show EDTA + Cu and DTPA + Cu induced membrane permeabilization, while these treatments did not increase lipid peroxidation.

Endodermal integrity is likely to be a critical factor controlling shoot concentrations when transport occurs via the apoplastic pathway. Increased translocation may result from apoplastic bypass flow caused by direct damage to the endodermis, or by reduced Cu-induced development of suberin lamellae [7]. In control and citric acid treatments, simulated endodermal damage greatly increases translocation to shoots, with the majority of the effect observed with less than 10% damage. Apoplastic bypass flow is therefore an important transport pathway, as previously theorized [87].

When transport occurs via the symplastic pathway, as is implicated by model simulations for chelate treatments, the endodermis no longer presents a barrier to solute transport. Increased endodermal damage therefore reduces symplastic flow, and consequently the transport of chelated solutes through the root for translocation to shoots. Experiments have found the barrier effect of the endodermis to be negated in chelator treatments [7]. The simulations presented here provide further support for the existence of a symplastic transport pathway for chelated metals.

## Appendix

**Table A1:** Sensitivity coefficients for global, grouped and specific parameter comparison sets analyzed using Latin Hypercube Sampling.

Comparison Set	Parameter ^†^	*C*_cortex_	*C*_endodermis_	*C*_xylem_	*C*_leaf_	Translocation
**Global Parameters**	*D*_a_	0.346 ***	0.057 ***	0.175 ***	0.207 **	0.183 *
	*D*_s_	0	0.004	0.004	0.035	0.035
	*v*_factor_	0	0.001	0.019	0.001	0
	*K*_L_	0.261 ***	0.076 **	0.011	0.242*	0.357 *
	α	0.009	0.003	0	0.006	0.006
	*P*_m_	0.280 ***	0.823 ***	0.756 ***	0.508 ***	0.416 ***
	*EDF*	0.105 **	0.034	0.035	0.001	0.002
**Parameter Groups**	*K*_pm_	0.444 **	0.433 **	0.432 **	0.325	0.325
Hydraulic conductivity: intact endodermis	*K*_xl_	0.049	0.049	0.048	0.141	0.141
	*K*_sym_	0.014	0.014	0.014	0	0
	K_cw_	0.01	0.01	0.01	0.012	0.012
	*K*_cb_	0.483	0.494	0.495	0.522	0.522
Hydraulic conductivity: damaged endodermis	*K*_pm_	0.019 ***	0.026	0.026	0.162	0.162
	*K*_xl_	0.081	0.076	0.076	0.246 *	0.244 *
	*K*_sym_	0.058	0.064 *	0.062	0.09	0.09
	*K*_cw_	0.000 ***	0.002	0.002	0.08	0.077
	*EDF*	0.841 ***	0.831 ***	0.834 ***	0.422 ***	0.427 ***
*D*_a_	*D*_acortex_	0.950 ***	0.931 ***	0.491 ***	0.430 **	0.183
	*D*_aendo_	0.006	0.002	0.070 *	0.046	0.083
	*D*_axylem_	0.005	0.043	0.054	0.118	0.099
	*D*_aleaf_	0.004 *	0.007	0.009	0.036	0.061
	*D*_s_	0.036 **	0.017 *	0.376 **	0.370 *	0.575 *
*D*_s_	*D*_scortex_	0.004 ***	0.009 ***	0.206 ***	0.158 ***	0.505 ***
	*D*_sendo_	0.090 *	0.199	0.157	0.154	0.059
	*D*_sleaf_	0.017	0.023 *	0.000 *	0	0.001
	*D*_acortex_	0.882 ***	0.757 ***	0.634 ***	0.656 ***	0.366 ***
	*D*_axylem_	0.007 **	0.012 ***	0.002 **	0.033 *	0.069
*K*_L_	*K*_Lcortex_	0.963 ***	0.905 ***	0.893 ***	0.322 ***	0.678
	*K*_Lendo_	0.006	0.06	0.023	0.163	0.082
	*K*_Lxylem_	0.007	0.024	0.043 ***	0.227 *	0.074 **
	*K*_Lleaf_	0.005	0.003	0.039	0.037 **	0.018 ***
	v_factor_	0.02	0.008	0.002 **	0.251 *	0.147 *
Permeability and dispersivity	*P*_membrane_	0.883 ***	0.931 ***	0.732 ***	0.724 ***	0.734 ***
	*P*_tonoplast_	0.005 *	0.005	0.008	0.008 *	0.008
	α_cortex_	0.001	0.005	0.004	0.005 *	0.004
	α_xylem_	0.01	0.002	0.04	0.041 **	0.039
	α_leaf_	0.101	0.057	0.217	0.222	0.215
**Specific Parameters**	*D*_acortex_	0.398 ***	0.115	0.046 ***	0.006	0.000 *
	*D*_scortex_	0.077	0.146	0.24	0.293	0.281
	*K*_Lcortex_	0.242 ***	0.006	0.056	0.055	0.018 *
	*EDF*	0.02	0.001	0.000 ***	0.004	0.006
	*P*_membrane_	0.263 **	0.733 ***	0.658 ***	0.643 ***	0.696 ***

* *p* < 0.05; ** *p* < 0.01; *** *p* < 0.001; ^†^ *D*_a_—Apoplastic diffusion; *D*_s_—Symplastic diffusion; *v*_factor_—Flow velocity; *K*_L_—Sorption coefficient; α—Dispersivity; *P*_m_—Permeability (plasma membrane and tonoplast); *EDF*—Endodermal damage fraction; *K*_pm_, *K*_xl_, *K*_sym_, *K*_cw_, *K*_cb_—Hydraulic conductivity of the plasma membrane, xylem, symplast, cell wall and casparian band, respectively; *D*_acortex_, *D*_aendo_, *D*_axylem_, *D*_aleaf_—Apoplastic diffusion in the cortex, endodermis, xylem and leaf, respectively; *D*_scortex_, *D*_sendo_, *D*_sleaf_—Symplastic diffusion in the cortex, endodermis and leaf, respectively; *K*_Lcortex_, *K*_Lendo_, *K*_Lxylem_, *K*_Lleaf_—Sorption coefficient in the cortex, endodermis, xylem and leaf, respectively; *P*_membrane_, *P*_tonoplast_—Permeability of the plasma membrane and tonoplast, respectively; α_cortex_, α_xylem_, α_leaf_—Dispersivity in the cortex, xylem and leaf, respectively.

**Table A2:** Summary of Cu speciation (% total Cu) for amendment treatments. Speciation was modeled using VisualMINTEQ v2.53.

Treatment	[Cu^2+^]	[CuHPO_4__(aq)_]	[Cu-Citrate^−^]	[CuEDTA^2−^]	[CuDTPA^3−^] + [CuHDTPA^2−^] + [Cu_2_DTPA^−^]
Control	68.7	20.4	1.1	n.a.	n.a.
Citric acid (0.157 mM)	20.4	5.5	70.23	n.a.	n.a.
Citric acid (2 mM)	0.5	0.17	96.6	n.a.	n.a.
EDTA (0.157 mM)	0.64	0.18	0.087	98.8	n.a.
DTPA (0.157 mM)	0.012	n.a.	n.a.	n.a.	86.5 + 7.5 + 6

n.a. = not applicable. Additional minor species are not shown.

**Table A3:** Membrane permeability of Cu, amendment ligands and complex species estimated using *K*_ow_ and *K*_DMPC/water_.

Species	Log *K*_ow_	Relative *P*_m_ (*K*_ow_ Basis) *	Log *K*_oc_^†^	Relative *P*_m_ (*K*_oc_ Basis) *	Log *K*_DMPC/water_^‡^	Relative *P*_m_ (*K*_DMPC/water_ Basis) *
Cu	−1.11 ^a^	1.00000	0.77	1.000	−1.111	1.0000
Citric acid	−1.64 ^b^	0.28684	0.48	0.500	−3.0164	0.0121
CuCitric	−2.19 ^a^	0.06774	0.18	0.211	−3.5694	0.0029
EDTA	−3.86 ^c^	0.00157	−0.72	0.028	−2.052	0.1007
CuEDTA	−5.03 ^a^	0.00009	−1.36	0.006	−3.225	0.0058
DTPA	−4.9 ^b^	–	−1.29	–	−1.909	–
CuDTPA	−5.22 ^a^	0.00005	−1.46	0.004	−2.219	0.0496

***** Estimated using reported aqueous diffusivities assuming membrane diffusivity to be proportional to aqueous diffusivity; ^†^ Estimated using the equation (log *K_oc_* = 0.544log *K_ow_* + 1.377) of Lyman, Reehl and Rosenblatt [88]; ^‡^ Estimated using structural fragment pseudoregression coefficients of Vaes *et al*. [89]; a Molinspiration Property Calculator, Molinspiration Cheminformatics, Slovensky Grob, Slovak Republic, 2011; b [90]; c [91], KowWin^®^ v1.67, Syracuse Research Corporation, Syracuse NY, 2000.
